# Delirium prevalence and management in general wards, emergency departments, rehabilitation centres and nursing homes in Germany, Austria and Switzerland (DACH countries): A secondary analysis of a worldwide point prevalence study

**DOI:** 10.1016/j.ijnsa.2025.100309

**Published:** 2025-02-10

**Authors:** Florian Schimböck, Lars Krüger, Magdalena Hoffmann, Marie-Madlen Jeitziner, Heidi Lindroth, Keibun Liu, Peter Nydahl, Rebecca Von Haken, Matthias Thomas Exl, Sibylle Fischbacher, WDAD-DACH study group

**Affiliations:** a Institute of General Practice, University Hospital of Schleswig-Holstein/Kiel University, Kiel, Germany; b Project and Knowledge Management/Care Development intensive care, Care Directorate, Heart and Diabetes Center NRW, University Hospital of the Ruhr University Bochum, Bad Oeynhausen, Germany; c Research Unit for Safety and Sustainability in Healthcare, c/o Division of Plastic, Aesthetic and Reconstructive Surgery, Department of Surgery, Medical University of Graz, Graz, Austria; d Department of Intensive Care Medicine, University Hospital Bern, Inselspital, University Bern, Bern, Switzerland; e Institute of Nursing Science (INS), Department of Public Health (DPH), Faculty of Medicine, University of Basel, Basel, Switzerland; f Division of Nursing Research, Department of Nursing, Mayo Clinic, Rochester, MN, USA; g Center for Aging Research, Regenstrief Institute, School of Medicine, Indiana University, Indianapolis, IN, USA; h Center for Innovation and Implementation Science, School of Medicine, Indiana University, Indianapolis, IN, USA; i Non-Profit Organization ICU Collaboration Network (ICON), Tokyo, Japan; j Nursing Research, University Hospital of Schleswig-Holstein, Kiel, Germany; k Institute of Nursing Science and Development, Paracelsus Medical University, Salzburg, Austria; l Department of Surgery, University Hospital Mannheim, Heidelberg, Germany; m Department of Neurosurgery, Inselspital, Bern University Hospital, University of Bern, Bern, Switzerland; n Graduate School for Health Science, University of Bern, Bern, Switzerland; o Department of Intensive Care Medicine, Community Hospital Zurich, Stadtspital, Zurich, Switzerland

**Keywords:** Delirium, Encephalopathy, Nurses, Prevalence, Protocol

## Abstract

**Background:**

Delirium is a common neuropsychiatric syndrome associated with an increased risk of mortality and length of stay. Current delirium prevalence, assessments and management practices of delirium in DACH countries are unknown.

**Objective:**

To examine the delirium point prevalence, assessment, management practices in general wards, emergency departments, rehabilitation centres and nursing homes in DACH countries.

**Design:**

Secondary data analysis from a prospective, cross-sectional, worldwide one-day point delirium prevalence study (registered in the German Registry for Clinical Trials, DRKS00030002)

**Setting(s):**

General hospital wards, emergency departments, rehabilitation centres and nursing homes excluding operating rooms, ambulatory, high acuity, intermediate care and intensive care units.

**Participants:**

In total, 172 wards (majority were general wards; 91.3 %, *n* = 157) from Germany, Austria and Switzerland participated.

**Methods:**

Descriptive analysis of a 39-questions online survey with aggregated routine patient and facility data completed by healthcare professionals, administrators, and researchers on World Delirium Awareness Day, March 15th, 2023. Data on delirium prevalence were collected at 8:00 A.M. and P.M. (± 4 h). Use of delirium assessments, delirium awareness structures, presence of care protocols, and barriers to delirium management structures were reported.

**Results:**

Overall delirium prevalence was 7.1 % (*n* = 143/2,028) at 8:00 A.M. and 7.2 % (*n* = 133/1,842) at 8:00 P.M. There was no statistically significant difference between the delirium prevalence assessed with valid (*p* = .770) or non-valid assessment (*p* = .643). The most frequent delirium assessments were the Nursing Delirium Screening Scale (16.3 %, *n* = 28/172), the Confusion Assessment Method (15.7 %, *n* = 27/172) and the Delirium Observation Screening Scale (9.3 %, *n* = 16/172). The most reported interventions to provide delirium awareness and education were “delirium is mentioned in handovers” (53.5 %, *n* = 92/172), “availability of delirium experts” (51.2 %, *n* = 88/172) and “delirium education during the last year” (48.3 %, *n* = 83/172). An existing delirium management protocol was present in 76.7 % (*n* = 132/172) of participating wards. The most frequently reported barriers to delirium care were “shortage of staff” (45.3 %, *n* = 78/172), “patients who are difficult to assess” (32.6 %, *n* = 56/172), and “communication gaps between professions” (29.1 %, *n* = 50/172). As non-pharmacological interventions, “mobilization” (92.4 %, *n* = 159/172), “pain management” (87.8 %, *n* = 151/172), and “adequate fluids” (83.7 %, *n* = 144/172) were mostly reported.

**Conclusions:**

Delirium is a frequent complication in patients in DACH countries. More than three quarter of participating wards reported a delirium management protocol suggesting awareness of published guidelines and best practice recommendations. Improved staffing, education on delirium assessment, and interprofessional communication could be helpful to improve the usage of valid delirium assessments and addressing barriers to delirium management.

**What is already known**

• Worldwide, every fifth to sixth institutionalized patient is assessed as being delirious.
• Point prevalence data about delirium in Germany, Austria and Switzerland are scarce.
• Every delirium should be avoided, as it has serious consequences for the patients, relatives, and healthcare professionals.

Alt-text: Unlabelled box

**What this paper adds**

• This secondary analysis identified a delirium point prevalence of about 7 % with no statistically significant difference between valid and non-valid delirium assessments in German speaking countries.
• There was a statistically significant lower delirium prevalence when wards indicated to assess patients generally thrice per day in comparison to wards using all other frequency types.
• Most of participating wards reported an existing delirium management protocol and more than half were using valid delirium assessments, especially the Nursing Delirium Screening Scale and the Confusion Assessment Method.

Alt-text: Unlabelled box

## 1 Background

Delirium is an acute and fluctuating neuropsychiatric syndrome, which is characterized by attention deficits and cognitive impairments accompanied by abnormal arousal and perceptual disturbances (Wilson et al., 2020). It can be triggered by an acute stressor, such as infection, dehydration, surgery, or prolonged immobility (Wilson et al., 2020). Delirium is often associated with adverse outcomes, including increased mortality, length of stay, functional decline, falls, distress in patients and caregivers, and long-term cognitive decline of inpatients in hospitals, nursing homes, or rehabilitation centres (Aung Thein et al., 2020; Goldberg et al., 2020; Williams et al., 2020). It impacts communication, decision-making and quality of life of patients (Logan, 2018). Despite these adverse outcomes and burden, delirium is often unrecognized and underdiagnosed by nurses and physicians (Bellelli et al., 2015; Clegg et al., 2011).

In adult medical inpatients, worldwide delirium prevalence ranges from 9 % to 34 % (Gibb et al., 2020; Koirala et al., 2020). Further results were found in Switzerland with 8.9 % in surgical patients, 4.8 % in medical patients and overall 4.6 % in wards (Schiess et al., 2024). In a point prevalence study in Italy, delirium prevalence was reported as 22.9 % of medical wards in acute hospitals (Bellelli et al., 2015), and comparable to data from Ireland (Ryan et al., 2013). In nursing homes, a delirium prevalence from 1.4 % to 70.3 % (Komici et al., 2022) and in rehabilitation centres or post-acute settings between 9 % to 26 % (Kiely et al., 2003; Marcantonio et al., 2003) were reported.

Until now, prevalence data about delirium in Germany (D), Austria (A) and Switzerland (CH) (DACH countries) as German speaking countries in general wards, emergency departments, rehabilitation centres and nursing homes are rare.

The primary aim of this secondary data analysis of a worldwide point prevalence study was therefore to indicate the prevalence in delirium of patients in general hospital wards, emergency departments, rehabilitation centres and nursing homes in DACH countries. Secondary aims were to identify the used delirium assessments, delirium awareness structures, presence of delirium protocols, barriers to delirium management practices, non-pharmacological and pharmacological interventions.

## 2 Methods

The reporting of this secondary data analysis follows the Strengthening the Reporting of Observational Studies in Epidemiology (STROBE) Statement (ESM Table E1) (von Elm et al., 2008).

### 2.1 Ethical considerations

The Institutional Review Boards of the DACH countries approved the main study, which was also registered in the German Registry.

### 2.2 Design, settings, and population

This is a secondary data analysis of the WDAD 2023 Delirium Survey for the DACH countries and includes data of general wards, emergency departments, rehabilitation centres and nursing homes in the DACH countries. Following wards were excluded: high acuity, intermediate care and intensive care units. Data of the main study and secondary analysis for other countries were published elsewhere (Azizi et al., 2024; Byrnes et al., 2024; Lindroth et al., 2024; Nydahl et al., 2024).

### 2.3 Data collection

Data from the main study (Nydahl et al., 2024) was collected on the World Delirium Awareness Day (March 15th 2023) using a 39-questions online survey which is available online (https://www.wdad-study.center). The survey was developed, revised, and pre-tested with an international and interprofessional team of healthcare professionals and researchers. The final survey consisted of 14 sections. The questions on demographic characteristics included detail information of the country, sociodemographic data of participants, and institution/ward specific data such as the total number of beds of the institution, the institution type (e.g. university hospital, nursing home, rehabilitation centre), the discipline working on ward (e.g. medical/non-surgical, surgical, long term care, mixed), the ward type (e.g. emergency department, general ward), and patient age group (e.g. <17 years, 18–75 years, >75 years, mixed). The term “mixed” was defined as a ward that cares for a large range of patients, including paediatric, adult, and geriatric patients. Questions about the delirium assessment included, e.g., the use of valid and non-valid assessment and the assessment frequency. The survey also collected information on which healthcare professional primarily carried out the delirium assessment. Valid assessments were defined as tools that had been generally acknowledged in the literature as reliable and validated in different patient settings such as the Nursing Delirium Screening Scale (Nu-DESC) or the Confusion Assessment Method (CAM). Non-valid delirium assessments included personal judgement, none and other. The term “other” is defined as a combination of valid and non-valid delirium assessments. Delirium awareness structures are understood as structural measures and conditions for delirium prevention and treatment. The survey included delirium awareness structures such as the use of educational trainings, informational posters and pocket cards, non-pharmacological interventions such as mobilization, cognitive stimulation and verbal re-orientation. Further questions were about the presence of delirium-related protocols such as pain, nutrition and sleep management; pharmacological interventions such as the use of drugs, inclusion of pharmacologists and reporting in handovers, and barriers against sufficient delirium management such as lack of time to educate staff, lack of awareness and missing knowledge. All questions based on previous delirium surveys (Krotsetis et al., 2018; Nydahl et al., 2018). The delirium prevalence at 8:00 A.M. (± 4 h) in the morning and 8:00 P.M. (± 4 h) in the evening based on the clinically documented delirium which was extracted by healthcare professionals (e.g. nurses and physicians) who participated in the study. Only aggregate ward level data were collected. Therefore, patients assessed at 8:00 A.M. may or may not have been included in the 8:00 P.M. sample depending on their individual length of stay.

### 2.4 Statistical analysis

The delirium prevalence was given as the number of patients assessed positive for delirium divided by the total number of patients assessed for delirium. The descriptive analysis was based on calculation of the mean and standard deviation (SD), median and interquartile range (IQR), and frequencies and percentages. Differences between groups of continuous and categorical variables were analysed using chi-square tests. The level of significance was established as 95 % (*p*-value < 0.025) for two-tailed tests. For the calculation of the probability of using validated delirium assessments and to analyse factors associated with the presence of delirium awareness structures, odds ratios (OR) and 95 % confidence intervals (95 % CI) were calculated also with the chi-square test. Missing data were reported if applicable. All analyses were performed using SPSS for Windows 29 (IBM Corp. Armonk, New York, USA).

## 3 Results

A total of 172 wards in Germany (52.9 %, *n* = 91/172), Austria (22.1 %, *n* = 38/172) and Switzerland (25.0 %, *n* = 43/172) participated in the study. Most of the wards in all DACH countries were part of university hospitals (68.6 %, *n* = 118/172) and reported >1500 hospital beds. General wards in all DACH countries represented 91.3 % (*n* = 157/172) of participating sites. Surgical disciplines in all DACH countries accounted for 44.8 % of the wards (*n* = 77/172) and 30.2 % (*n* = 52/172) were classified as medical/non-surgical disciplines. The median number of beds per ward was 25 (IQR 20.8–30.0) (Table 1).

**Table 1:** Characteristics of participating institutions and wards.

	Total	Germany	Austria	Switzerland
**Survey respondents per country n (%)**				
Participating wards	172 (100.0)	91 (52.9)	38 (22.1)	43 (25.0)
**Type of institution n (%)**				
University hospital	118 (68.6)	66 (72.5)	38 (100.0)	14 (32.6)
Community hospital	23 (13.4)	5 (5.5)	0 (0.0)	18 (41.9)
Private hospital	19 (11.0)	14 (15.4)	0 (0.0)	5 (11.6)
University-related hospital	5 (2.9)	5 (5.5)	0 (0.0)	0 (0.0)
Rehabilitation centre	5 (2.9)	1 (1.1)	0 (0.0)	4 (9.3)
Nursing home	2 (1.2)	0 (0.0)	0 (0.0)	2 (4.7)
**Numbers of beds per institution n (%)**				
<250	17 (9.9)	5 (5.5)	0 (0.0)	12 (27.9)
<500	34 (19.8)	15 (16.5)	0 (0.0)	19 (44.2)
<750	20 (11.6)	20 (22.0)	0 (0.0)	0 (0.0)
<1000	17 (9.9)	8 (8.8)	0 (0.0)	9 (20.9)
<1500	19 (11.0)	17 (18.7)	0 (0.0)	2 (4.7)
>1500	65 (37.8)	26 (28.6)	38 (100.0)	1 (2.3)
**Type of discipline n (%)**				
Medical/non-surgical	52 (30.2)	25 (27.5)	12 (31.6)	15 (34.9)
Surgical	77 (44.8)	39 (42.9)	23 (60.5)	15 (34.9)
Palliative	2 (1.2)	1 (1.1)	0 (0.0)	1 (2.3)
Rehabilitation	4 (2.3)	1 (1.1)	0 (0.0)	3 (7.0)
Long term care	2 (1.2)	0 (0.0)	0 (0.0)	2 (4.7)
Mixed/general	21 (12.2)	14 (15.4)	2 (5.3)	5 (11.6)
Other	14 (8.1)	11 (12.1)	1 (2.6)	2 (4.7)
**Type of ward n (%)**				
Emergency department	5 (2.9)	3 (3.3)	1 (2.6)	1 (2.3)
General ward	157 (91.3)	85 (93.4)	37 (97.4)	35 (81.4)
Rehabilitation facility	3 (1.7)	0 (0.0)	0 (0.0)	3 (7.0)
Nursing home	3 (1.7)	0 (0.0)	0 (0.0)	3 (7.0)
Other	4 (2.3)	3 (3.3)	0 (0.0)	1 (2.3)

### 3.1 Delirium prevalence

Data of 2028 patients assessed for delirium at 8:00 A.M. and of 1842 patients at 8:00 P.M. were collected from the DACH countries. The overall delirium prevalence according to valid and non-valid assessments was 7.1 % (*n* = 143/2028) at 8:00 A.M. and 7.2 % (*n* = 133/1842) at 8:00 P.M. In those patients assessed with a valid delirium assessment, delirium was present in 7.3 % (*n* = 79/1083) at 8:00 A.M., and 7.0 % (*n* = 70/1005) at 8:00 P.M. In those patients assessed without valid delirium assessment, delirium was present in 6.8 % (*n* = 64/945) at 8:00 A.M. and in 7.5 % (*n* = 63/837) at 8:00 P.M. There was no statistically significant difference between patients assessed with valid delirium assessments versus non-valid delirium assessments at 8:00 A.M (*p* = .770) and 8:00 P.M. (*p* = .643). In Austria, the prevalence of delirium was highest with 10.4 % (*n* = 25/240) in the morning, and 9.8 % (*n* = 24/246) in the evening. The lowest delirium prevalence was measured in Germany with 4.9 % (*n* = 30/611) at 8:00 A.M. and 5.4 % (*n* = 28/523) at 8:00 P.M (Table 2).

**Table 2:** Delirium prevalence using valid delirium assessment.

	Morning: 8:00 A.M. ± 4 h		Evening: 8:00 P.M. ± 4 h	
	Total n	Delirious n (%)	Total n	Delirious n (%)
Delirium prevalence	1083	79 (7.3)	1005	70 (7.0)
**Countries**				
Germany	611	30 (4.9)	523	28 (5.4)
Austria	240	25 (10.4)	246	24 (9.8)
Switzerland	232	24 (10.3)	236	18 (7.6)
**Age groups**				
0–17 years	36	0 (0.0)	34	0 (0.0)
18–75 years	619	39 (6.3)	584	33 (5.7)
>75 years	173	19 (11.0)	114	14 (12.3)
Mixed	252	21 (8.3)	269	23 (8.6)
**Type of discipline**				
Medical/non-surgical	330	36 (10.9)	293	33 (11.3)
Surgical	537	25 (4.7)	502	22 (4.4)
Palliative	10	0 (0.0)	0	0 (0.0)
Rehabilitation	0	0 (0.0)	0	0 (0.0)
Long Term Care	30	2 (6.7)	30	2 (6.7)
Mixed/general	123	16 (13.0)	130	13 (10.0)
Other	53	0 (0.0)	50	0 (0.0)
**Type of ward**				
Emergency department	8	1 (12.5)	8	2 (25.0)
General ward	987	73 (7.4)	906	62 (6.8)
Rehabilitation facility	0	0 (0.0)	0	0 (0.0)
Nursing Home	41	3 (7.3)	44	3 (6.8)
Other	47	2 (4.3)	47	3 (6.4)

The prevalence of delirium was statistically significantly different between DACH countries in the morning (*p* = .003) but not in the evening (*p* = .074). There was a statistically significant lower delirium prevalence when wards indicated to assess patients generally thrice per day in comparison to wards using all other frequency types, both at 8:00 A.M. (2.0 %, *n* = 6/298 vs. 9.3 %, *n* = 73/785, *p* < .001) and at 8:00 P.M. (3.9 %, *n* = 11/279 vs. 8.1 %, *n* = 59/726, *p* = .020).

### 3.2 Delirium assessment

Of the 172 wards, 58.1 % (*n* = 100) used a valid delirium assessment. The most frequently used valid delirium assessments were the Nursing Delirium Screening Scale (Nu-DESC, 16.3 %, *n* = 28/172), the Confusion Assessment Method (CAM, 15.7 %, *n* = 27/172) and the Delirium Observation Screening Scale (DOSS, 9.3 %, *n* = 16/172). In total, 32.1 % of the wards (*n* = 52/162) assessed for delirium only in case of sudden changes of consciousness, and 21.6 % (*n* = 35/162) used an assessment frequency of three times per day. “Other” assessment frequency was reported by 25.3 % (*n* = 41/162) of the included wards, in Germany 4.9 % (*n* = 4/82), Austria 23.7 % (*n* = 9/38) and highest in Switzerland with 66.7 % (*n* = 28/42) (Table 3).

**Table 3:** Delirium assessment data reported by survey respondents.

	Total *n* = 172	Germany *n* = 91	Austria *n* = 38	Switzerland *n* = 43
**Presence of valid delirium assessment n (%)**				
Valid delirium assessment yes	100 (58.1)	50 (54.9)	28 (73.7)	22 (51.2)
Valid delirium assessment no	72 (41.9)	41 (45.1)	10 (26.3)	21 (48.8)
**Valid delirium assessment n (%)**				
Nu-DESC	28 (16.3)	15 (16.5)	13 (34.2)	0 (0.0)
CAM	27 (15.7)	9 (9.9)	1 (2.6)	17 (39.5)
DOSS	16 (9.3)	5 (5.5)	8 (21.1)	3 (7.0)
CAM-ICU	10 (5.8)	10 (11.0)	0 (0.0)	0 (0.0)
Psychiatric consult	8 (4.7)	3 (3.3)	4 (10.5)	1 (2.3)
CAM-IMC	5 (2.9)	5 (5.5)	0 (0.0)	0 (0.0)
4-AT	3 (1.7)	2 (2.2)	0 (0.0)	1 (2.3)
CAP-D	2 (1.2)	0 (0.0)	2 (5.3)	0 (0.0)
DSM-V criteria	1 (0.6)	1 (1.1)	0 (0.0)	0 (0.0)
**Non-valid delirium assessment n (%)**				
Personal/clinical judgement	19 (11.0)	16 (17.6)	0 (0.0)	3 (7.0)
None	12 (7.0)	12 (13.2)	0 (0.0)	0 (0.0)
Other	41 (23.8)	13 (14.3)	10 (26.3)	18 (41.9)
**Delirium assessment frequency n (%)**				
Only in case of sudden changes of consciousness	52 (32.1)	28 (34.1)	16 (42.1)	8 (19.0)
Thrice per day (24 h)	35 (21.6)	31 (37.8)	0 (0.0)	4 (9.5)
Twice per day (24 h)	14 (8.6)	4 (4.9)	10 (26.3)	0 (0.0)
Only at admission	10 (6.2)	7 (8.5)	3 (7.9)	0 (0.0)
Once per day (24 h)	9 (5.6)	7 (8.5)	0 (0.0)	2 (4.8)
More than thrice per day (24 h)	1 (0.6)	1 (1.2)	0 (0.0)	0 (0.0)
Other	41 (25.3)	4 (4.9)	9 (23.7)	28 (66.7)
Missing data	10 (5.8)	9 (9.8)	0 (0.0)	1 (2.3)

Abbreviations: Nu-DESC = Nursing Delirium Screening Scale; CAM= Confusion Assessment Method; DOSS= Delirium Observation Screening Scale; CAM-ICU= Confusion Assessment Method for the Intensive Care Unit; CAM-IMC= Confusion Assessment Method for the Intermediate Care; 4AT= 4 ‘A's Test (Arousal, Attention, Abbreviate Mental Test – 4, Acute change); CAP-D= Cornell Assessment of Pediatric Delirium; DSM-V criteria= Diagnostic and Statistical Manual of Mental Disorders, Fifth Edition; Other= Valid and non-valid assessments combined.

Of all respondents, 10.5 % (*n* = 18) reported a non-valid delirium assessment. Half of these respondents (50,0 %, *n* = 9) were using “personal/clinical judgement”, 11.1 % (*n* = 2) no assessment for delirium identification, and 38.8 % (*n* = 7) reported that they have a valid delirium assessment, but think it is not the right one for their discipline. In all three countries, nurses were reported as the profession primarily responsible for delirium assessment (79.7 %, *n* = 137/172). In Germany, 14.3 % (*n* = 13/91) stated that multiple professions are responsible for delirium assessment, while in Austria and Switzerland this was the case in only 0.0 % (*n* = 0/38) and 2.3 % (*n* = 1/43) respectively.

There was a statistically significant association between presence of valid delirium assessments and delirium management protocols (*p* < .001). In case the wards had an existing delirium management protocol, there was a 7.65 times higher chance that a valid delirium assessment was used to non-valid delirium assessment (OR 7.65; 95 % CI 3.34–17.51).

In case wards offered different delirium awareness structures, there was a 13.31 times higher chance that a valid delirium assessment was used to non-valid delirium assessment (OR 13.31; 95 % CI 3.79–46.76). Other strongly associated factors for the use of valid delirium assessments were “pocketcards for delirium assessment/management” (OR 5.34; 95 % CI 2.51–11.31; *p* < .001), “delirium experts known by the staff” (OR 2.50; 95 % CI 1.34–4.67; *p* = .004) and “delirium flyer for the staff” (OR 2.33; 95 % CI 1.23–4.43; *p* = .009) (ESM Table E2).

### 3.3 Delirium awareness structures, management protocols and barriers

The top three reported interventions to provide delirium awareness and education were “delirium is mentioned in handovers” (53.5 %, *n* = 92/172), “availability of delirium experts” (51.2 %, *n* = 88/172) and “delirium education during the last year” (48.3 %, *n* = 83/172) (ESM Table E3).

The most frequently reported delirium-related written protocols included “pain management” (89.5 %, *n* = 154/172), “delirium management” (76.7 %, *n* = 132/172), and “nutrition management” (57.6 %, *n* = 99/172) (ESM Table E4).

The most reported barriers for implementation and/or use of evidence-based interventions in delirium care were “shortage of staff” (45.3 %, *n* = 78/172), “patients who are difficult to assess” (32.6 %, *n* = 56/172), and “communication gaps between professions” (29.1 %, *n* = 50/172). In contrast, the three least mentioned barriers were “leadership does not support” (4.1 %, *n* = 7/172), “lack of pharmacological interventions” (3.5, *n* = 6/172), and “no cost/resources for promoting at department” (2.3 %, *n* = 4/172) (Table 4).

**Table 4:** Reported barriers to delirium management.

	Total *n* = 172	Germany *n* = 91	Austria *n* = 38	Switzerland *n* = 43
**Barriers against implementation and/or use of evidence-based strategies are … n (%)**				
Shortage of staff	78 (45.3)	37 (40.7)	22 (57.9)	19 (44.2)
Patients who are difficult for assessment	56 (32.6)	24 (26.4)	16 (42.1)	16 (37.2)
Communication gaps between professions	50 (29.1)	30 (33.0)	8 (21.1)	12 (27.9)
Lack of time to educate and train staff	50 (29.1)	30 (33.0)	8 (21.1)	12 (27.9)
Lack of delirium knowledge	46 (26.7)	30 (33.0)	7 (18.4)	9 (20.9)
We have no barriers, delirium is regularly assessed	45 (26.2)	18 (19.8)	11 (28.9)	16 (37.2)
Lack of delirium awareness	32 (18.6)	19 (20.9)	2 (5.3)	11 (25.6)
Other problems are more challenging	30 (17.4)	19 (20.9)	9 (23.7)	2 (4.7)
Missing attitude (delirium is not important)	21 (12.2)	16 (17.6)	1 (2.6)	4 (9.3)
No appropriate scores for delirium assessment	18 (10.5)	13 (14.3)	1 (2.6)	4 (9.3)
Not enough motivated staff	16 (9.3)	12 (13.2)	1 (2.6)	3 (7.0)
Lack of non-pharmacological interventions	12 (7.0)	8 (8.8)	0 (0.0)	4 (9.3)
Interprofessional conflicts	8 (4.7)	3 (3.3)	0 (0.0)	5 (11.6)
Leadership does not support	7 (4.1)	5 (5.5)	0 (0.0)	2 (4.7)
Lack of pharmacological interventions	6 (3.5)	4 (4.4)	1 (2.6)	1 (2.3)
No cost/resources for promoting at the department	4 (2.3)	2 (2.2)	2 (5.3)	0 (0.0)
Other barriers*	149 (86.6)	82 (90.1)	30 (78.9)	37 (86.0)

⁎ Other barriers: Physicians do not know the protocols; No continuous presence of doctors in the ward; Delirium prevalence is low in my setting; Overload of documentation.

### 3.4 Delirium prevention and treatment interventions

The top three non-pharmacological interventions that participants reported at least once per shift were “mobilization” (92.4 %, *n* = 159/172), “pain management” (87.8 %, *n* = 151/172), and “adequate fluids” (83.7 %, *n* = 144/172). In comparison, the use of “activities in patient groups” (12.8 %, *n* = 22/172), “physical restraints" (7.0 %, *n* = 22/172), and “animal assisted therapy” (3.5 %, *n* = 6/172) were the least frequently reported interventions (Table 5).

**Table 5:** Non-pharmacological interventions used as delirium prevention and/or treatment.

	Total *n* = 172	Germany *n* = 91	Austria *n* = 38	Switzerland *n* = 43
**Total amount of non-pharmacological delirium interventions on wards**				
Mean	12.15	11.57	15.08	10.79
Standard deviation	4.62	4.84	2.95	4.32
**Non-pharmacological delirium interventions present on wards n (%)**				
Mobilization (i.e. sitting on the edge of bed or more during daytime)	159 (92.4)	81 (89.0)	37 (97.4)	41 (95.3)
Pain management	151 (87.8)	77 (84.6)	36 (94.7)	38 (88.4)
Adequate fluids	144 (83.7)	68 (74.7)	37 (97.4)	39 (90.7)
Provision of vision, hearing and mobility aids	143 (83.1)	71 (78.0)	37 (97.4)	35 (81.4)
Non-disturbed sleep (i.e. reduction of noise and light)	143 (83.1)	73 (80.2)	36 (94.7)	34 (79.1)
Cognitive stimulation (i.e. newspaper, TV, music)	138 (80.2)	75 (82.4)	35 (92.1)	28 (65.1)
Provision of day- and night rhythm	137 (79.9)	71 (78.0)	34 (89.5)	32 (74.4)
Verbal re-orientation	134 (77.9)	69 (75.8)	37 (97.4)	28 (65.1)
Open/liberal visiting times (daytime)	121 (70.3)	59 (64.8)	34 (89.5)	28 (65.1)
Sharing or communication patient information about delirium	100 (58.1)	47 (51.6)	30 (78.9)	23 (53.5)
Avoidance of bladder tubes and catheters	94 (54.7)	36 (39.6)	31 (81.6)	27 (62.8)
Family information	90 (52.3)	44 (48.4)	28 (73.7)	18 (41.9)
Multiprofessional team rounds	90 (51.3)	45 (49.5)	29 (76.3)	16 (37.2)
Ear plugs and sleep glasses	74 (43.0)	48 (52.7)	13 (34.2)	13 (30.2)
Special trained delirium and dementia carer	62 (36.0)	27 (29.7)	28 (73.7)	7 (16.3)
Family engagement	59 (34.3)	28 (30.8)	21 (55.3)	10 (23.3)
Sitters (beside the patient for longer time)	59 (34.3)	24 (26.4)	26 (68.4)	9 (20.9)
Bed border	55 (32.0)	44 (48.4)	4 (10.5)	7 (16.3)
Ground-leveled beds	40 (23.3)	11 (12.1)	21 (55.3)	8 (18.6)
Going outside the ward (i.e. garden, sunlight)	29 (16.9)	18 (19.8)	3 (7.9)	8 (18.6)
Multiprofessional daily goals	28 (16.3)	13 (14.3)	10 (26.3)	5 (11.6)
Activities in patient groups (i.e. singing, eating, exercises)	22 (12.8)	13 (14.3)	5 (13.2)	4 (9.3)
Physical restraints	12 (7.0)	7 (7.7)	1 (2.6)	4 (9.3)
Animal assisted therapy	6 (3.5)	4 (4.4)	0 (0.0)	2 (4.7)
Other interventions	126 (73.3)	77 (84.6)	12 (31.6)	37 (86.0)

Other interventions= Multimodal stimulation (basale stimulation, aromatherapy, fidget blankets), Orientation aids (calendar, clocks, photos); Bed exit alarms (floor alarm mats); Validation therapy; Specialized unit for cognitive impairment.

The top three pharmacological interventions were: “quetiapine” (47.1 %, *n* = 81/172), “risperdone” (36.6 %, *n* = 63/172), and “reduction of potentially delirogenous drugs” (34.3 %, *n* = 59/172).

Survey respondents reported that pharmacological management across units “depends on the specific symptoms of each patient's delirium” (73.3 %, *n* = 126/172), “is reported in handovers” (49.4 %, *n* = 85/172), and “includes a psychiatrist or delirium specific liaison team” (40.7 %, *n* = 70/172). In addition, 30.8 % (*n* = 53/172) stated that they “discuss the pharmacological management with patients”, 20.9 % (*n* = 36/172) “include pharmacologists”, and 16.9 % (*n* = 29/172) stated that the pharmacological management “is a general approach, including a few pharmacological agents”. ESM Table E5 provides details about the used drugs and pharmacological interventions.

## 4 Discussion

In this secondary analysis of data from WDAD 2023 in general wards, emergency departments, rehabilitation centres and nursing homes of DACH countries, we observed an overall delirium prevalence of 7.1 % (*n* = 143/2028) in the morning and 7.2 % (*n* = 133/1842) in the evening. In total, 58.1 % (*n* = 110/172) of wards were using a valid delirium assessment and 76.7 % had an existing delirium management protocol. The top three reported interventions to provide delirium awareness and education were “delirium is mentioned in handovers” (53.5 %, *n* = 92/172), “availability of delirium experts” (51.2 %, *n* = 88/172) and “delirium education during the last year” (48.3 %, *n* = 83/172). The most reported barriers for sufficient delirium care were “shortage of staff” (45.3 %, *n* = 78/172), “patients who are difficult to assess” (32.6 %, *n* = 56/172), and “communication gaps between professions” (29.1 %, *n* = 50/172). The top three non-pharmacological interventions were “mobilization” (92.4 %, *n* = 159/172), “pain management” (87.8 %, *n* = 151/172), and “adequate fluids” (83.7 %, *n* = 144/172).

### 4.1 Delirium prevalence

The overall delirium prevalence in DACH countries seems to be lower than in previous results in general wards in Italian hospitals (22.9 %) (Bellelli et al., 2015), emergency departments of different countries (up to 36.0 %) (Wang et al., 2024), rehabilitation centres in USA (up to 26.0 %) (Kiely et al., 2003; Marcantonio et al., 2003) or nursing homes of different countries (up to 70.3 %) (Komici et al., 2022). In our analysis, 91.3 % of the wards were general wards in hospitals. Emergency departments (2.9 %) rehabilitation centres (1,7 %) and nursing homes (1,7 %) were underrepresented and did not participate from all DACH countries. The time intervals between the individual publications may have an influence on the prevalence of delirium, as new guidelines and recommendations may have led to changes in practice in the meantime.

Considering the results of other secondary analysis of the WDAD 2023 data, the prevalence is comparable. The WDAD study team in USA reported a delirium prevalence in general wards of 10 % at 8:00 A.M and 12 % at 8:00 P.M. (Lindroth et al., 2024). Ornago et al. (2024) described a delirium prevalence of 9.6 % at 8:00 A.M. and 10.4 % at 8:00 P.M. in general wards of Italian hospitals.

Interestingly, German wards had around 50 % less delirious patients (4.9 %) in the morning than Austrian (10.4 %) and Swiss wards (10.3 %). These results are comparable to a delirium prevalence of 4.2 % in patients after heart surgery in Germany (Hulde et al., 2024) and 4.6 % in hospital wards of a point prevalence study in Switzerland (Schiess et al., 2024). Systematic reviews showed a delirium prevalence of 10.8 % in a German university hospital (Gao et al., 2022; Zipprich et al., 2020), 13.6 % in emergency departments in European countries (Chen et al., 2022) and 15.0 % in different countries (Wang et al., 2024). Like in our analysis, patients in general wards were in focus of the most studies. Zipprich et al. (2020) discussed the influence of different types of wards and prevention strategies like urinary catheters and changes of rooms/wards on their low delirium prevalence. The difference can be explained by different non-pharmacological interventions, different delirium assessments and the effective use of delirium protocols that might have had an influence on the low prevalence in our study. In other hypotheses, the influence of patient related risk factors like age and further treatment on delirium were discussed (Schiess et al., 2024). “Who seeks much, finds much”- this is a use of speech that everyone knows. But: interestingly our analysis indicated a statistically significantly lower delirium prevalence in wards which assessed delirium thrice per day compared to other frequency types. Maybe the more frequent use will also lead to a safer application of the assessment and less false results. Another possibility is that the use of assessments in patient settings for which they have not been validated has an impact on the accuracy of the results. Future research should include more patient-related outcomes like anxiety or pain (Blume et al., 2021) to enable further analyses and conclusions. Moreover, we could not find a statistically significant difference in delirium prevalence between the use of valid and non-valid assessments. A possible explanation is that participating facilities have a great interest in delirium prevention and have already developed a specific expertise in delirium diagnostics comparable with the use of valid delirium assessments. This hypothesis could also offer an interesting approach for future research projects.

### 4.2 Delirium assessment

In Germany and Austria, the Nu-DESC was mostly used while the CAM was most prevalent in Switzerland. Internationally, the 4 'A's Test (4AT), the Nu-DESC (Ho et al., 2022; Jeong et al., 2020; Penfold et al., 2024), the CAM and the DOSS (De and Wand, 2015) are mostly described in the context of general hospital wards. These differences can be explained by country-specific cultures. In 2007, Hasemann et al. (2007) published the German translation of the DOSS and the CAM which since than have been used in the “Basel Delirium Management Program” in Switzerland. Similarly, in Germany, Lutz et al. (2008) published the German translation of the Nu-DESC in 2008 and recommended the Nu-DESC for routine delirium assessment. Further, Lin et al. (2023) considered the Nu-DESC to be easier to use for nurses in daily practice than the CAM.

A few respondents reported that their used delirium assessment is maybe not the right one for their discipline. Interestingly, we found out that ten Germany-based wards stated that they use the Confusion Assessment Method for the Intensive Care Unit (CAM-ICU) in general wards. De and Wand (2015) identified also studies in wards in which the CAM-ICU was used. Hence, delirium assessments were not implemented on a population-specific basis, but “top-down” on an institution-wide basis.

In our study, only about 50 % of all wards used a valid delirium assessment. Aldecoa et al. (2024) recommend the use of a valid delirium assessment at least once per day. In this context, Penfold et al. (2024) discussed a possible positive influence through a prevention initiative, but also reasons for insufficient screening like less knowledge of the staff, lack of user-friendliness, and no population-specific delirium assessment. Against the backdrop of staff shortages, this could also apply to our study. In future, more awareness for delirium assessment should be reached (Koirala et al., 2020) taking into account our results that wards with an existing delirium awareness structures have a higher chance to use a valid delirium assessment.

We found that almost 80 % of assessments were performed by nurses in DACH countries. These results are similar to other findings reported from Germany (Nydahl et al., 2018) and other countries (Lin et al., 2023; Penfold et al., 2024). However, the diagnostic process of delirium should be an interprofessional one. Local guidelines and educational programs can support and improve interprofessional collaboration in the delirium assessment and diagnosis (Lin et al., 2023; Nydahl et al., 2018; Schimböck et al., 2024). Future research may examine how novel technologies such as automated measurement, artificial intelligence can improve delirium prediction and assessment in clinical care.

### 4.3 Delirium awareness structures, management protocols and barriers

The participating wards reported different strategies to support delirium awareness. Delirium was, among others, mentioned in handovers with additionally trainings. Both aspects are also part of delirium prevention programs and helpful in daily practice (Deeken et al., 2021; Lee et al., 2020).

Our mostly reported barriers in delirium management were similar to the findings of Bianchi et al. (2024). The authors also reported e.g. shortages of staff, challenges in assessment of patients as confounders, communication challenges and conflicts between healthcare professionals, lack of time for education and lack of knowledge about delirium. Bianchi et al. (2024) concluded that extended training for healthcare professionals should be implemented. Trainings should ideally be interprofessional and lead to the formation of local delirium working groups (Schimböck et al., 2024). Such groups could be helpful to initiate specific quality improvement projects (e.g. creation of one minute wonder (Krüger et al., 2022), provide an interprofessional team approach and to overcome the described barriers (Schimböck et al., 2024). Future studies may examine the effects of delirium awareness structures and educational strategies on delirium-related knowledge.

### 4.4 Delirium prevention and treatment interventions

An intensive awareness, multiple further education and staff training, non-pharmacological interventions and the use of drugs with a reduced trigger can prevent delirium (Aldecoa et al., 2024; Kim et al., 2022; Kudchadkar et al., 2022). Burton et al. (2021) also indicated a positive effect of multi-component non-pharmacological treatment. An exploratory network analysis showed that especially re-orientation, sleep hygiene and cognitive stimulation may also reduce the incidence of delirium (Burton et al., 2021). In our study, mobilization, pain management, and adequate fluids were mostly named as non-pharmacological interventions in delirium prevention and management. Additionally, the provision of vision, hearing and mobility aids, non-disturbed sleep (e.g. reduction of noise and light) and cognitive stimulation (e.g. newspaper, TV, music) were named by >80 % of the participating wards. Well realised and similar non-pharmacological interventions were also reported in the analysis of the WDAD data of Ireland (Azizi et al., 2024), Italy (Ornago et al., 2024) and the USA (Lindroth et al., 2024). Furthermore, these results belong to the general national and international recommendations (Aldecoa et al., 2024; Hauss et al., 2021) and show a successful implementation in practice. Nydahl et al. (2018) reported similar findings in DACH countries in context of mobilization which was mostly named as prevention and therapy of delirium management (77.3 %, *n* = 432). The national German speaking early mobilization network (Nydahl et al., 2020) may have had a positive effect of mobilization awareness here in recent years. For example, free monthly newsletters via email including summarized evidence about, among others, early mobilization are available. Furthermore, there is a national (Hermes et al., 2022) and updated international guideline (Aldecoa et al., 2024) and an algorithm (Ottens et al., 2024) for delirium prevention and management available and probably used to develop local guidelines and/or protocols.

Guideline recommendations for delirium management include the avoidance or minimization of antipsychotics and benzodiazepines (Aldecoa et al., 2024; Hermes et al., 2022; Soiza and Myint, 2019). Despite these recommendations, quetiapine and risperidone use were reported by 47.1 % and 36.6 % respectively in the present study. It is promising that more than a third mentioned the reduction of potentially delirogenous drugs as management strategy. It is recommended that future implementation studies and quality improvement projects focus on aligning delirium care with the best practice recommendations for the pharmacological management. Furthermore, future research should focus on delirium prevention strategies and the development of tailored approaches for different healthcare settings.

## 5 Strength and limitations

A strength of our secondary data analysis is an overview of delirium prevalence and management in the DACH countries. The 2023 WDAD survey provided a structured and pre-tested framework that could serve as a benchmark for measuring future progress.

Our secondary data analysis has also limitations. First, a recruitment bias is highly likely through the methodology of the main study. It is likely that more institutions or wards that are already interested in the delirium topic or have established a delirium program have participated in the survey than institutions or wards where delirium needs more attention. This is also indicated by participation of only few emergency departments, rehabilitation centres and nursing homes. Future surveys may address this limitation by proactively recruiting institutions or wards. Second, the survey was designed with the intention of facilitating ease of completion and thus, detailed patient data were not collected, such as age, biological sex, or co-morbidities. For this reason, it was not possible to map whether the assessment instruments used were also validated for the respective setting. The calculated delirium prevalence may therefore differ from the actual delirium prevalence in practice, as the assessments used may not have been validated for the respective group of patients. It is therefore not known what other factors may have contributed to delirium. Recording was done in two measure points (morning and evening) and due to different assessment intervals in studies, it is difficult to compare these results with others. Potentially the staff have not mastered the assessments used, so that there may have been false positive or false negative results.

## 6 Conclusions

Delirium remains a challenge for healthcare in general wards in hospitals, emergency departments, rehabilitation centres and nursing homes in German speaking countries. More than 75 % of participating wards had an existing delirium management protocol suggesting awareness of published guidelines and best practice recommendations. Furthermore, it was shown that with an assessment frequency of three times a day, the prevalence is lower than with other types of assessment frequency. Additional staff, continuous education in delirium assessment, interprofessional collaboration, patient communication, family engagement, improvement of non-pharmacological prevention and treatment strategies, and minimization of pharmacological interventions could be helpful to optimize evidence-based delirium care. This study could be the starting point to take actions to guide future educational trainings, increase delirium awareness, initiate quality improvement projects to overcome existing barriers and to strengthen delirium management in DACH and other countries.

## Funding sources

This secondary data analysis was financed by own funds.

## CRediT authorship contribution statement

**Florian Schimböck:** Conceptualization, Methodology, Data curation, Formal analysis, Writing – original draft, Writing – review & editing. **Lars Krüger:** Conceptualization, Methodology, Data curation, Formal analysis, Writing – review & editing. **Magdalena Hoffmann:** Conceptualization, Writing – original draft, Writing – review & editing. **Marie-Madlen Jeitziner:** Conceptualization, Methodology, Data curation, Formal analysis, Supervision, Writing – original draft, Writing – review & editing, Project administration. **Heidi Lindroth:** Conceptualization, Writing – original draft, Writing – review & editing. **Keibun Liu:** Conceptualization, Writing – original draft, Writing – review & editing. **Peter Nydahl:** Conceptualization, Methodology, Data curation, Formal analysis, Supervision, Writing – original draft, Writing – review & editing, Project administration. **Rebecca Von Haken:** Conceptualization, Writing – original draft, Writing – review & editing. **Matthias Thomas Exl:** Conceptualization, Writing – original draft, Writing – review & editing. **Sibylle Fischbacher:** Conceptualization, Writing – original draft, Writing – review & editing.

## Declaration of competing interest

HL is funded by the NIA 1AGK23076662-02, serves as a board member and 2023 conference co-chair for the American Delirium Society, and serves as the web committee chair for the American Thoracic Society, Nursing Assembly. She has received royalties for keynote addresses at nursing conferences in 2022 and received a travel scholarship to attend the 2023 DECLARED conference in Sydney, Australia. RVH received support from OrionPharma to attend meetings and/or travel. No conflicts are reported by FS, LK, MH, MMJ, KL, PN, MTE, and SF.

## Appendix B Supplementary materials

Image, application 1
